# Monte Carlo investigation of the characteristics of radioactive beams for heavy ion therapy

**DOI:** 10.1038/s41598-019-43073-1

**Published:** 2019-04-25

**Authors:** Andrew Chacon, Mitra Safavi-Naeini, David Bolst, Susanna Guatelli, Daniel R. Franklin, Yuma Iwao, Go Akamatsu, Hideaki Tashima, Eiji Yoshida, Fumihiko Nishikido, Atsushi Kitagawa, Akram Mohammadi, Marie-Claude Gregoire, Taiga Yamaya, Anatoly B. Rosenfeld

**Affiliations:** 1 Centre for Medical Radiation Physics, University of Wollongong, Wollongong, NSW 2522 Australia; 2 Australian Nuclear Science and Technology Organisation (ANSTO), Lucas Heights, NSW Australia; 3 Brain and Mind Centre, University of Sydney, Sydney, NSW Australia; 4 Illawarra Health and Medical Research Institute, University of Wollongong, Wollongong, NSW 2522 Australia; 5 Faculty of Engineering and IT, University of Technology Sydney, Ultimo, NSW 2007 Australia; 6 National Institute of Radiological Sciences (NIRS), National Institutes for Quantum and Radiological Science and Technology, 4-9-1 Anagawa, Inage-ku, Chiba 263-8555 Japan

**Keywords:** Biophysics, Biological physics

## Abstract

This work presents a simulation study evaluating relative biological effectiveness at 10% survival fraction (RBE10) of several different positron-emitting radionuclides in heavy ion treatment systems, and comparing these to the RBE10s of their non-radioactive counterparts. RBE10 is evaluated as a function of depth for three positron-emitting radioactive ion beams (^10^C, ^11^C and ^15^O) and two stable ion beams (^12^C and ^16^O) using the modified microdosimetric kinetic model (MKM) in a heterogeneous skull phantom subject to a rectangular 50 mm × 50 mm × 60 mm spread out Bragg peak. We demonstrate that the RBE10 of the positron-emitting radioactive beams is almost identical to the corresponding stable isotopes. The potential improvement in PET quality assurance image quality which is obtained when using radioactive beams is evaluated by comparing the signal to background ratios of positron annihilations at different intra- and post-irradiation time points. Finally, the incidental dose to the patient resulting from the use of radioactive beams is also quantified and shown to be negligible.

## Introduction

Heavy ion therapy (HIT) is a relatively new cancer treatment modality, with several facilities operating or under construction around the world^1–3^. A monoenergetic heavy ion beam deposits most of its energy within a narrow depth range - known as the Bragg Peak - with the peak dose depth determined by the beam energy, ion species and target composition^4,5^. Irradiation of the entire target volume is achieved using a range of particle energies, either via a passive scatterer or a raster-scanned spot beam with varying energy. Due to the narrow depth range of the Bragg peak, together with minimal lateral scattering and the high relative biological effectiveness (RBE) of heavy ions, HIT delivers a highly conformal therapeutic dose to the target volume with a much lower entrance dose than is possible with photon therapy. HIT achieves a lower entrance dose compared to proton therapy, although unlike proton therapy, some dose is delivered beyond the distal edge of the target volume due to the fragmentation tail.

The precision of HIT makes it particularly useful for treating deeply-situated tumours while minimising damage to adjacent healthy tissue^4,6–8^. However, due to the large dose gradients, deviations between the treatment plan and the delivered dose distribution can result in significant adverse effects on healthy tissue, particularly if the treatment region is in the proximity of an organ at risk (OAR). Accurate real-time measurement of spatial dose distribution during irradiation will provide a mechanism for closed-loop control over the treatment process, minimising errors between the treatment plan and the actual delivered dose.

During HIT, a fraction of the ions in the beam will undergo nuclear inelastic collisions. Fragmentation of nuclei either from the primary beam or in the target and entrance path result in the production of a range of stable and radioactive nuclei^6^. Some of these fragments are positron-emitting radionuclides, which continue to travel a short distance in the target before coming to a stop, where they eventually decay. Measuring of the distribution of these secondary positron-emitting fragments offers a unique opportunity for nonÂinvasive, real-time and/or offline quality assurance (QA) in heavy ion therapy via positron emission tomography (PET)^9–16^.

A large number of annihilation photons must be detected in order to obtain a PET image of sufficient quality for useful treatment QA. The cross-sections for inelastic ion collisions depend on several parameters, including incident ion species and energy, and the density and composition of the target^17^. These factors determine the mix of fragments produced, which, in turn, determines the number and distribution of positron-emitting radionuclides resulting from each beam spill. To improve image quality, several authors have proposed the use of positron-emitting radioactive nuclei (such as ^11^C, ^15^O or ^10^C) as the primary particle in the heavy ion beam. Most primary particles will survive intact to decay via positron emission at their stopping point, corresponding to the location of the Bragg peak. Therefore, for radioactive beams, the spatial distribution of the stopping points of primary particles is the dominant component of the PET image, while positron-emitting target and beam fragments making up a secondary component.

Beamlines capable of producing beams of radioactive ion species such as ^11^C, ^10^C and ^15^O with sufficient dose rates and beam purity for therapeutic use are currently under development at the National Institutes for Quantum and Radiological Science and Technology (NIRS, QST) in Japan and other facilities around the world^18–23^.

In order to perform proper treatment planning with positron-emitting radioactive beams, and to understand how their use will impact image-based QA, it is necessary to address three key research questions:

1. How does the relative biological effectiveness (RBE) of polyenergetic radioactive beams vary as a function of depth within a spread out Bragg peak, and how does this compare to the corresponding stable ion species?
2. What quantitative differences are expected between the maps of positron annihilation resulting from treatment with stable and positron-emitting radioactive ion beams, and how will these impact the use of PET images as an intra-treatment or post-treatment QA mechanism? and finally,
3. What additional dose will be received by the patient if a positron-emitting radioactive beam is used instead of a stable beam?

In this work, simulations of a simple treatment plan (consisting of a flat biological dose in a rectangular-prismatic primary treatment volume inside a human skull phantom) are performed for five primary nuclei (three positron-emitting and two stable) using the Geant4 Monte Carlo toolkit. The values of RBE_10_ (RBE at 10% survival fraction) are estimated across a range of depths along the beam path (in the entrance, SOBP and tail regions) using Kase’s modified microdosimetric kinetic model (MKM)^24–26^. The validity of using Monte Carlo simulations to evaluate RBE using the MKM has previously been established by Bolst *et al*.^27,28^; however, to our knowledge, this is the first time that this approach has been applied to estimate the RBE_10_ of a polyenergetic radioactive beam. The method can easily be extended to other homogeneous or heterogeneous targets and heavy ion species, and is a convenient and cost-effective alternative to *in vitro* experiments.

Monte Carlo simulation-based 2D maps of positron yield obtained in a skull phantom using a spread out Bragg peak (with the same flat biological dose (in Gy(RBE)) delivered throughout the planned treatment volume) are compared across all beam types. The distribution of positron production in the target volumes, as measured during the beam-off periods during irradiation of the phantom with the radioactive and corresponding stable heavy ion beams were measured, and the resulting signal to background ratios (SBRs) estimated. The chosen physics models in the simulation are validated via experimental work conducted at NIRS’s HIMAC facility.

Finally, the additional dose to the patient resulting from the use of radioactive beams is estimated to determine whether it poses any significant risk to the patient compared to the use of a stable ion beam.

The remainder of this paper is organised as follows. A summary of key related work, including a description of the modified MKM which is adopted in this paper, is presented in Section 2. Details of the Monte Carlo simulations, including the phantom, physical and biophysical models used and the experimental validation of the selected physics models, the implementation of a pseudo-clinical beamline and treatment plan for stable and their corresponding radiactive ion beams are discussed in Section 3. Simulation results and analysis of the RBE_10_ values of stable and radioactive beams, the resulting positron yield maps and the incidental dose resulting from the use of the radiactive beams are presented and discussed in Section 4. Conclusions and proposed future work are presented in Section 5.

## Related Work

The use of positron-emitting radioisotopes for heavy ion therapy has been investigated by a number of authors. In 2001, Urakabe *et al*. demonstrated that a positron-emitting ^11^C scanned spot beam could be directly used as the therapeutic agent^29^. However, the estimate of RBE_10_ used to obtain a flat biological dose was based on an extrapolation of previously-reported results for ^12^C in water, which was assumed to extend to human tissue^30^. Iseki *et al*. at NIRS used low-intensity monoenergetic ^10^C probe beams with between 10 ^4^ and 10 ^5^ particles per spill to estimate the depth of the therapeutic ^12^C beam’s Bragg peak, while keeping the dose received during the range measurement under 100 mGyE (a few percent of therapeutic dose)^31^. RBE of the radioactive beam was estimated via simulation using the one-dimensional HIBRAC beam transportation code from Sihver *et al*. combined with Kanai’s RBE model^30,32,33^. However, this work only considered monoenergetic ^11^C ion beams, and ignored the effects of low-LET fragmentation products, which resulted in an overestimation of the RBE for ^11^C. Augusto *et al*. used the FLUKA Monte Carlo toolkit to investigate the use of ^11^C beams either alone or in conjunction with ^12^C^34^. It was found that for beams with equivalent energy per nucleon incident on the same water phantom, ^11^C and ^12^C beams produce very similar fragmentation products, with the main differences being the relative yield of helium ions and several boron isotopes. While this study demonstrated the potential of using ^11^C in heavy ion therapy, it only considered monoenergetic beams of ^11^C at a fixed depth (100 mm) in a homogeneous water phantom. The composition of the phantom, the isotope and the specific beam energy are important factors affecting the fragmentation processs and the spatial distribution of positron-emitting nuclei which results^35,36^.

These works demonstrate the potential for using positron-emitting beams both for radiotherapy and for range verification. However, in order to conclusively establish their clinical utility, it is necessary to quantify their RBE and evaluate the quality of the resulting PET image in a clinically relevant configuration, through the use of heterogenous tissue-equivalent phantoms and polyenergetic ion beams.

Relative biological effectiveness (RBE) is an empirically-derived ratio which can be used to predict the physical dose of a specific type of radiation which will result in the same cellular survival fraction as a reference dose (typically a 200 keV X-ray beam)^37,38^. The complex dependencies of RBE on the energy and type of radiation, as well the location of the target and the specific tissue types present, require the use of biophysical methods for accurate theoretical estimation of RBE^39–41^. The Microdosimetric Kinetic Model (MKM), proposed by Hawkins *et al*., is a widely-used method for estimating RBE in which the microdosimetric spectrum (*f*(*y*)) is measured through the use of a tissue-equivalent proportional counter (TEPC)^24^. It was subsequently extended by Kase *et al*. to relate the saturation-corrected dose-mean lineal energy (${\bar{y}}^{\ast }$) to the radiation sensitivity coefficient *α* of the linear quadratic model (LQM, measured in units of Gy^−1^ and Gy^−2^), such that the method can be applied to therapeutic heavy ion beams^25,26,42^. This *modified MKM* has been extensively validated for carbon ion therapy, and also extended to proton and helium ion therapy^25,26,42–44^.

The RBE_10_ for an ion beam, defined as the ratio of the physical dose from a 200 kVp X-ray beam required to achieve a cellular survival fraction of 10% (*D*_(10,*R*)_) to the ion beam dose resulting in the same cell survival fraction, can be derived using the microdosimetric spectra *f*(*y*), using (1), (2) and (3):

1 $${y}^{\ast }={y}_{0}^{2}\frac{\int (1-{e}^{-{(\frac{y}{{y}_{0}})}^{2}})\,f(y)dy}{\int yf(y)dy}$$

2 $$\alpha ={\alpha }_{0}+\frac{{\beta }_{0}}{\rho \pi {r}_{d}^{2}}{y}^{\ast }$$

3 $$RB{E}_{10}=\frac{2\beta {D}_{10,X \mbox{-} ray}}{\sqrt{{\alpha }^{2}-4\beta \,\mathrm{log}\,(0.1)}-\alpha }$$

For human salivary gland (HSG) tumour cells, the dose resulting in a survival fraction of 10%, *D*_(10,*R*)_ is 5 Gy for 200 kVp X-rays; the LQM radiation sensitivity coefficient values are *α*_0_ = 0.13 Gy^−1^ and *β*_0_ = 0.05 Gy^−2^. *ρ* and *r*_*d*_ are the density and the radius of the sub-cellular domain, and assumed to be 0.42 *μ*m and 1 g/cm^3^, respectively^25^.

In this work, RBE_10_ is estimated using an extension to the modified MKM proposed by Bolst *et al*., whereby the mean path length $< {l}_{path} >$ of the charged particles that cross the sensitive volume is introduced to account for the directionality of the radiation field when deriving the microdosimetric spectra *f*(*y*) in a non-spherical sensitive volume, as opposed to the average chord length used in isotropic fields^27,28^.

Although estimates of the RBE_10_ for radioactive beams have been reported previously, these have been calculated using simplified analytic models with parameters interpolated/extrapolated from limited experimental data from beams of stable isotopes in homogeneous targets^45,46^. The assumption that the RBE of radioactive ion species can be estimated from its stable analog has not been previously demonstrated in the literature.

## Method

All Monte Carlo simulations were performed using the Geant4 toolkit (version 10.2.p03)^47,48^. The hadronic physics models used in the simulations are listed in Table 1, while electromagnetic interactions were modelled using the standard Geant4 option 3 physics constructor (G4EmStandardPhysics_option3). The hadronic physics processes and models are listed in Table 1.

**Table 1:** Hadron physics models used in all simulations.

Interaction	Energy Range	Geant4 Model/Package
Radioactive Decay	All energies	G4RadioactiveDecayPhysics
Particle Decay	All energies	G4Decay
Hadron Elastic	All energies	G4HadronElasticPhysicsHP
Ion Inelastic	0–110 MeV	Binary Light Ion Cascade
	>100 MeV	QMDModel
Neutron Capture	0–20 MeV	NeutronHPCapture
Neutron Inelastic	0–20 MeV	NeutronHPInelastic
	_>_20 MeV	Binary Cascade
Proton Inelastic	0–9.9 GeV	Binary Cascade
EM Interactions	All energies	G4EmStandardPhysics_option3

Section 3.1 details the methods used to experimentally validate the Geant4 simulation. The phantoms used in the simulations are described in detail in Section 3.2. In Sections 3.3, 3.4 and 3.5, the implementation of the modified MKM for the evaluation of the RBE_10_ of pseudo-clinical, polyenergetic carbon and oxygen beams and their corresponding radioactive beams is described. Lastly, Section 3.6 describes a simulation study which examines the yield of different positron-emitting radionuclides during and after the irradiation of a skull phantom with radioactive and corresponding stable beams and introduces the metric used for the evaluation of the quality of the resulting annihilation maps.

### Experimental validation of the physics models

To validate the Monte Carlo physics models, several simulations evaluating depth-dose profiles and positron-emitting radionuclide yield were performed and compared with measurements obtained from equivalent physical experiments.

All experiments were performed at the Heavy Ion Medical Accelerator in Chiba (HIMAC), Japan, with the stable ion beams produced at the primary beam course, and the radioactive ion beams at the secondary beam course^19,23^. The peak energies of the non-radioactive ^12^C and ^16^O ion beams, as measured at the beamline nozzle, were 290 MeV/u and 400 MeV/u, respectively with an energy spread of *σ* = 0.2%. The peak energies of the radioactive ^11^C and ^15^O ion beams were 330 MeV/u and 290 MeV/u, respectively, each with an energy spread of *σ* = 5%. The nominal transverse diameter of all beams was 2 mm full width at half maximum (FWHM).

#### Depth-dose relationship

**Figure 1:**
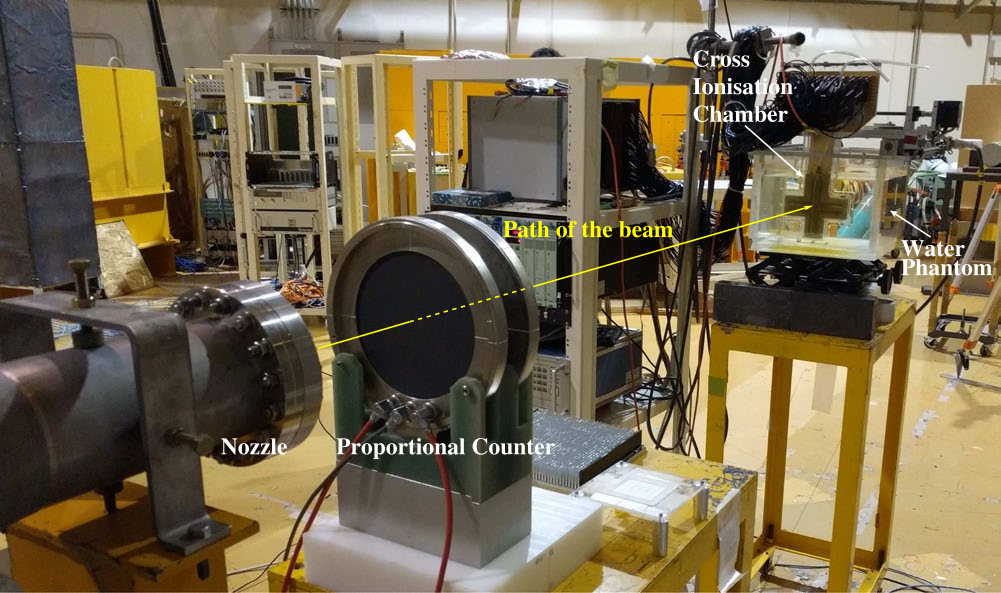
The experimental configuration used to estimate the depth-dose profile of the stable ion beams in water, at the primary beam course (HIMAC, Japan); the radioactive beams were produced at the secondary beam course (not shown in this image).

The experimental configuration shown in Fig. 1 was used to compare the experimental and simulation-based depth-dose curves. The deposited energy was measured using a pre-calibrated cross ionisation chamber (IC) with a sensitive volume of 36 mm^3^, inside a 300 × 300 × 300 mm^3^ water phantom^23^. The IC was encased within a 0.5 mm PMMA casing and moved along the path of the beam using a motorised stage, with an accuracy of 10 *μ*m. The energy deposited within the ionisation chamber at each point along the beam was normalised to the energy deposited at the entrance (i.e. at the front of the phantom). All depth measurements were converted to water equivalent depth.

For the simulation study, each beam was modelled using a monoenergetic incident beam with a Gaussian energy distribution, with the same peak energies and spreads as for the HIMAC beamlines. The simulated beams entered the water phantom perpendicular to its front surface (see Table 2), with an air gap of 2.5 m between the beamline nozzle and the phantom surface as per the corresponding experimental configuration. The energy deposited was scored in the water phantom using 1 mm^3^ voxels and summed over a 36 mm^3^ volume equivalent to the sensitive volume of the ionisation chamber used throughout the experimental measurements. Energy deposited in the sensitive volume (as a function of depth) was normalised to value observed at the entrance plateau.

**Table 2:** Phantom compositions.

Phantom Name	Phantom material	Dimensions
PMMA phantom	PMMA	100 × 100 × 300 mm^3^
Water phantom	Water	250 × 250 × 250 mm^3^
Skull phantom	Bone	250 × 250 × 10 mm^3^
	Brain Tissue (modelled as muscle)	250 × 250 × 240 mm^3^

#### Positron-emitting fragmentation product yield

The hadronic physics models of Geant4, including the Quantum Molecular Dynamics (QMD) ion hadronic inelastic scattering and Radioactive Decay physics models, were validated by comparing the simulated and experimentally estimated yields of ^11^C, ^10^C and ^15^O, the three dominant positron-emitting radionuclides generated during irradiation of a 100 × 100 × 300 mm^3^ PMMA phantom by monoenergetic ^12^C and ^16^O beams with energies of 290 MeV/u and 400 MeV/u, respectively.

**Figure 2:**
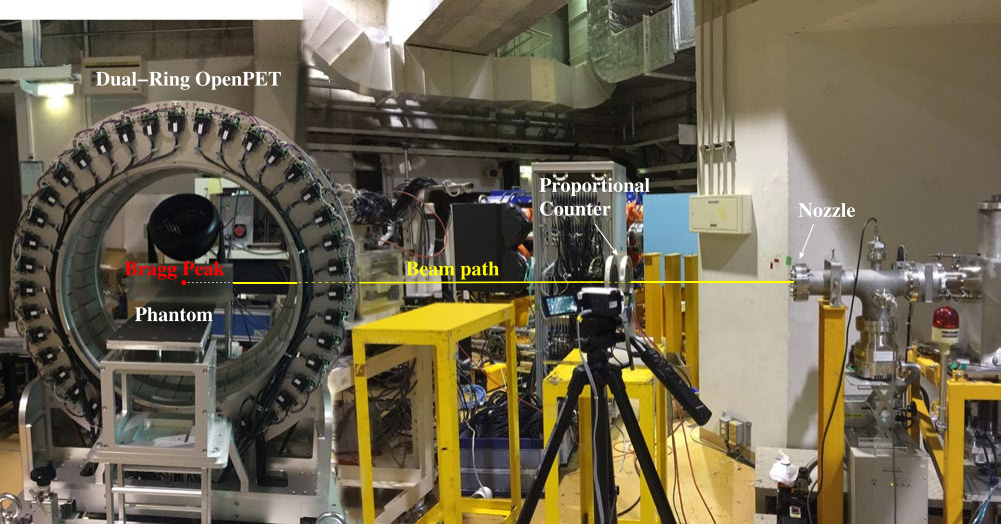
The experimental configuration used in HIMAC, Japan, to validate the QMD ion hadronic inelastic scattering model used in the simulations. The phantom is positioned within the field of view (FOV) such that the calculated location of the Bragg peak (indicated by a red dot) is placed at the centre of the field of view (CFOV).

The experimental configuration is shown in Fig. 2. The phantom was positioned such that the expected location of the Bragg peak was aligned with the centre of the field of view in the OpenPET scanner^14^. 20 spills were used, with a beam intensity of 1.0 × 10^9^ particles per second (pps). In each spill, the beam was on for 1.9 seconds and off for 1.4 seconds. List-mode PET data were collected intra-spill, and for 36 minutes after the final spill. Dynamic (4D) images were reconstructed using the 3D ordinary Poisson ordered-subset-expectation-maximisation algorithm (3D-OP-OSEM) with 1.5 × 1.5 × 1.5 mm^3^ voxels. Temporal frame lengths were chosen so as to be able to observe decay over several half-lives of ^11^C, ^10^C and ^15^O. Yields of each positron-emitting radionuclide were estimated by fitting the parameters of a simple analytical model to the observed time-activity curves (TACs). Total activity as a function of time *t* in a volume with initial activities of ^11^C, ^10^C and ^15^O of *A*_0,*C*11_, *A*_0,*C*10_ and *A*_0,*O*15_, respectively, is given by

4 $${A}_{total}(t)={A}_{0,C11}{e}^{-\mathrm{ln}\mathrm{(2)}t/{T}_{C11}}+{A}_{0,C10}{e}^{-\mathrm{ln}\mathrm{(2)}t/{T}_{C10}}+{A}_{0,O15}{e}^{-\mathrm{ln}\mathrm{(2)}t/{T}_{O15}}$$

where *T*_*C*11_, *T*_*C*10_ and *T*_*O*15_ are the respective half-lives of ^11^C, ^10^C and ^15^O. Total activity is measured as a function of time across the build-up and Bragg peak region, defined as the region from the point at which the dose profile has risen 5% above the entrance plateau to the point after which the profile is below 5% of the peak value.

The individual initial activities for each radionuclide are then estimated for both the simulation results and the experimental data by fitting the model to the observed curve.

For the simulation studies, monoenergetic ^12^C and ^16^O beams were directed perpendicularly to the surface of a simulated 10 × 10 × 30 cm^3^ PMMA with an air gap of 1.75 m between the beamline nozzle and the phantom surface, matching the experimental configuration. Density, mean excitation, ionisation potential and dimensions of simulated phantoms were chosen to match those used in the experiment. The spatio-temporal distributions of positron-emitting nuclei, positron production and annihilation were recorded with a scoring volume resolution of 1.5 mm, corresponding to the voxel dimensions in the experimental PET images. Simulated yield profiles were convolved with a Gaussian filter, with its FWHM equal to the estimated OpenPET spatial resolution (3.5 mm)^14^.

### Phantom geometry

The phantoms used in the simulation were rectangular prisms with compositions as listed in Table 2. All material compositions were based on data from the National Institute of Standards and Technology (NIST) database^49^.

### Estimation of RBE_10_ for a pseudo-clinical SOBP

To evaluate and compare the RBE_10_ of polyenergetic stable and positron-emitting radioactive beams, sensitive 1 mm × 1 mm × 10 *μ*m volumes were defined every 100 *μ*m along the path of the beam. The lineal energy deposition spectrum in each volume for all interactions (*f*(*y*)) was stored and used to calculate the *RBE*_10_ at that point, using (3) (equivalent results for monoenergetic carbon and oxygen ion beams with the energies listed in Supplementary Table S1 are presented in Supplementary Table S2). A correction factor 1.05 were used to account for the difference in stopping power and density of water relative to brain tissue.

A simple variance analysis method was used to estimate a sufficient number of primary particles to use in the simulations. *M* test simulations were conducted, each with *N* primary particles, with RBE estimated for each simulation and the mean and standard deviation (SD) calculated across the *M* simulations. The standard deviation should approach zero as *N* tends to infinity; therefore, in this experiment, *N* was progressively doubled with a fixed value of *M* = 50 until the ratio of standard deviation to mean was less than an arbitrary threshold of 1%. This analysis suggested that *N* = 10^7^ would be sufficient to get a good estimate of RBE (95% probability of the estimated RBE being within ±2% of the true RBE).

### Carbon

The spectrum for the simulated carbon beams was generated using an experimentally-validated model of the passively-scattered ^12^C beamline at HIMAC, which is known to produce a flat biological dose across a 60 mm depth range^50^. The spectra of the positron-emitting radioactive beams (^10^C and ^11^C) were based on the ^12^C spectrum from this beamline, by determining the energies for which the Bragg peaks of monoenergetic radioactive ion beams where located at the proximal and distal edges of the desired SOBP, and linearly mapping the weights of the energies of the ^12^C SOBP spectrum to this range of energies. Finally, the SOBPs were compared and confirmed to both correspond to the planned depths.

### Oxygen

Currently a validated model of the ^16^O beamline does not exist. Therefore, generation of the 60 mm flat biological dose SOBP in the target depth range was achieved by performing monoenergetic Monte Carlo simulations of an ^16^O beam at a range of energies (177, 237, 297, 345 and 418 MeV/u), and evaluating the RBE_10_ as a function of depth for each energy using the modified MKM (see Section 3.3 and Supplementary Table S2). This RBE was used to convert the physical dose deposited in the simulations to an estimated biological dose for the 5 evaluated energies. Profiles were then generated for other intermediate energies by interpolating between the simulated values in increments of 1 MeV/u. Finally, the target flat biological dose was achieved by adjusting the weights of each of these profiles such that a flat biological dose rate of 5 Gy(RBE)/min was achieved within the target depth range. The spectra of the positron-emitting radioactive beam (^15^O) was based on the ^16^O spectrum, with energies scaled such that the SOBP was positioned in the desired depth range (as per carbon).

### Positron-emitting radionuclide yield study

The impact of using positron-emitting primary beams on interspill and post-irradiation image quality was evaluated by comparing the spatial distributions of positron decays observed in the simulation over several different intervals during treatment of the skull phantom. A simple treatment plan was designed for each primary particle type, aimed at producing a constant biological dose rate of ≈5 Gy(RBE)/min in a depth range of 78–138 mm within a skull phantom. A total of 1 × 10^9^ primary particles were used in each simulation. As for the experimental validation study, twenty spills were simulated, with the beam on for 1.9 seconds and off for 1.4 seconds.

The distributions of positron decays were acquired for each beam type between the first and second spill, during the first five inter-spill intervals, and in the five minutes following the final spill.

The contrast-to-noise ratios (CNRs) between the inside and outside of the proximal, distal and upper lateral edges of the SOBP are computed for each image. The CNR provides a metric for objectively comparing the specificity with which the irradiated region is delineated, and is defined as:

5 $$CNR=\frac{|{\mu }_{a}-{\mu }_{b}|}{\sqrt{({\sigma }_{a}^{2}+{\sigma }_{b}^{2}}}$$

where *μ*_*a*_ and *μ*_*b*_ are the mean signal amplitudes and *σ*_*a*_ and *σ*_*b*_ are the standard deviations of the image intensity in two regions *a* and *b* of the image^51,52^.

## Results and Discussion

### Physics model validation

**Figure 3:**
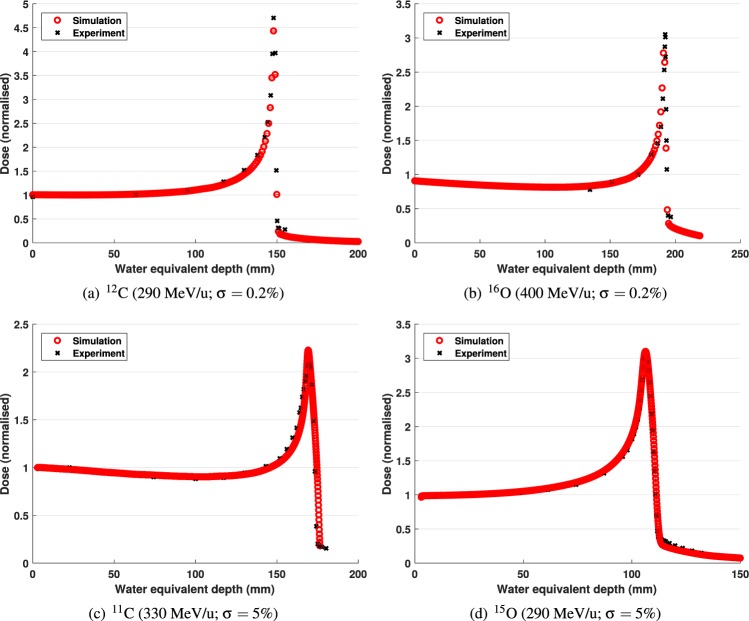
Experimental and simulated energy deposited in the sensitive volume plotted as a function of depth for the ^12^C, ^16^O, ^11^C and ^15^O ion beams. The deposited energy is normalised to value observed at the entrance plateau.

Experimental and simulation-based depth-dose curves are shown in Fig. 3; the difference between the locations of the Bragg peaks obtained from the simulated and the experimental ^12^C, ^16^O, ^11^C and ^15^O depth-dose profiles were 0.8 mm, 0.24 mm, 0.37 mm and 0.43 mm, respectively.

The experimental and simulation yields of ^10^C, ^11^C, and ^15^O produced during the irradiation of a PMMA phantom using a 290 MeV/u ^12^C beam and a 400 MeV/u ^16^O beam are expressed as a percentage of the total positron-emitting radionuclide yield and are listed in Table 3.

**Table 3:** Relative yields of positron-emitting nuclei in experiment and simulation.

Primary beam	Energy (MeV/u)	Isotope	Relative Yield (%)	
			Simulation	Experimental
^12^C	290	^11^C	80 ± 8	82 ± 9
		^10^C	5 ± 3	4 ± 2
		^15^O	15 ± 6	14 ± 8
^16^O	400	^11^C	44 ± 10	43 ± 10
		^10^C	7 ± 7	7 ± 5
		^15^O	49 ± 14	50 ± 10

The close agreement between the experimental and simulated normalised depth-dose profiles and the relative yield estimations of the positron-emitting fragments demonstrate the validity of the simulation model. The small differences between the experimental and simulated depth dose profiles for radioactive primary particles may be due to an underestimation of the initial energy spread, heterogeneity of the beryllium target leading to contamination with other fragments and systematic errors introduced by the ionisation chamber measurements.

### RBE and biological dose in Gy(RBE)

**Figure 4:**
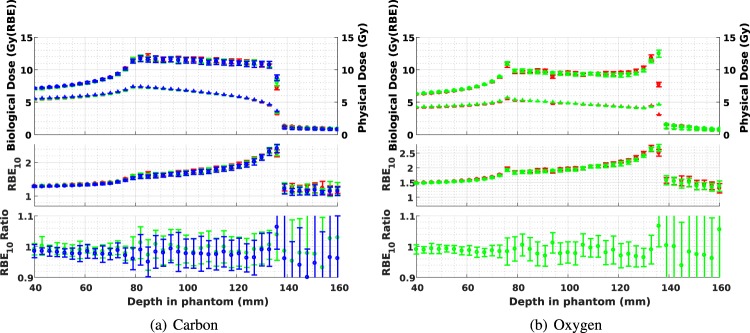
Biological dose, physical dose and RBE_10_ for positron-emitting radioactive beams, together with the ratio of radioactive-beam RBE_10_ to stable-beam RBE_10_, each shown as a function of depth within the phantom. The objective is a uniform dose within a 60 mm SOBP, from 78 to 138 mm depth. For carbon, ^12^C is shown in red, with ^11^C and the ratio of ^11^C: ^12^C shown in green, and ^10^C and the ratio of ^10^C:^12^C shown in blue. ^16^O is shown in red, while both ^15^O and the ratio of ^15^*O*: ^16^O is shown in green. All confidence intervals are 95% (two standard deviations).

Figure 4 presents a comparison of RBE_10_ as a function of depth for the positron-emitting radioactive beams and for the corresponding stable isotope beams (for clarity, RBE_10_ values are shown at depth increments of 3 mm; refer to Supplementary Spreadsheet 1 for a full list of RBE_10_ values evaluated at 100 *μ*m intervals for all ion species). In each case, the mean RBE_10_s of the stable and radioactive beams are well within each others’ 95% confidence interval. Radioactive-to-stable RBE_10_ ratios are also shown, with the mean values remaining very close to 1.0 in the entrance and SOBP. The larger confidence intervals in the tail region are due to very little energy being deposited beyond the end of the SOBP (as expected for heavy ion beams), resulting in significant statistical noise.

Table 4 lists numerical values of the RBE_10_ obtained at the entrance, the beginning, middle and end of the SOBP, and tail region for each beam type. The mean and standard deviations presented are calculated over 11 consecutive 100 *μ*m deep sample volumes along the beam path centred about the listed depth. In all cases, the mean RBE_10_s for the radioactive and corresponding stable ion beams are within one standard deviation of each other.

**Table 4:** Means and standard deviations of the RBE_10_ for each beam evaluated at five depths (entrance, start, middle and end of SOBP, and tail).

Region	Depth (mm)	^12^C RBE_10_		^11^C RBE_10_		^10^C RBE_10_		^16^O RBE_10_		^15^O RBE_10_	
		μ	σ	μ	σ	μ	σ	μ	σ	μ	σ
Entrance	50	1.32	0.0577	1.31	0.0646	1.30	0.0511	1.51	0.0455	1.50	0.0469
Start of SOBP	81	1.61	0.184	1.61	0.182	1.56	0.148	1.84	0.137	1.84	0.173
Middle of SOBP	111	1.80	0.202	1.79	0.199	1.76	0.235	2.05	0.163	2.05	0.190
End of SOBP	131	2.21	0.251	2.23	0.258	2.20	0.256	2.59	0.215	2.46	0.187
Tail	171	1.15	0.396	1.12	0.317	1.12	0.365	1.28	0.501	1.27	0.407

At each depth, RBE_10_ is evaluated in 11 adjacent sensitive volumes (every 100 *μ*m along the path of the beam) and the mean and standard deviation calculated.

The significance of this result is that it indicates that the evaluated radioactive ion beams are comparable to their non-radioactive counterparts in terms of relative biological effectiveness. Heavy ion therapy with any of the radioactive ion species examined in this study should be feasible, with only minimal changes to the current treatment planning algorithms required to account for the small differences in RBE_10_.

### Positron yield

**Figure 5:**
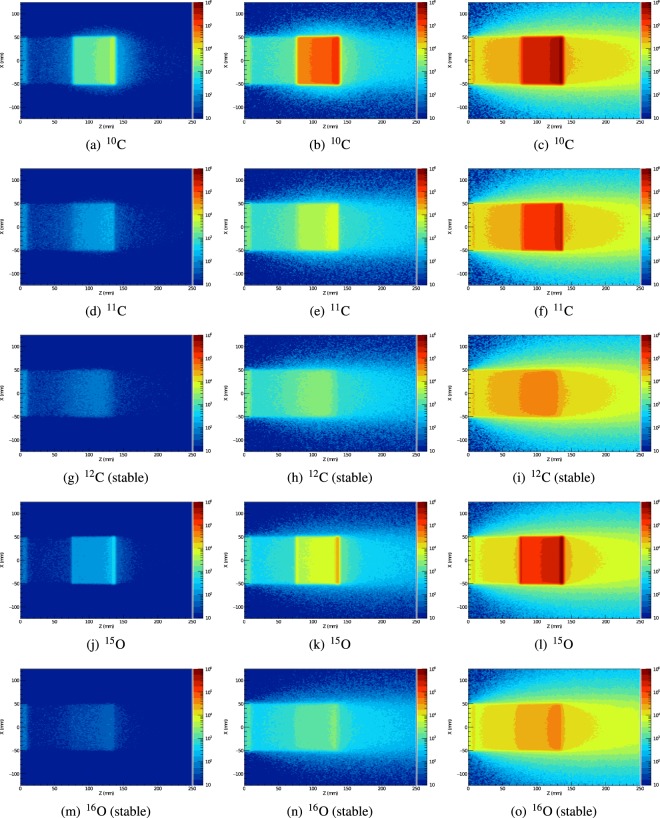
2D positron annihilation maps resulting from 5 Gy(RBE) irradiation of the skull phantom, during and after irradiation: after 1 of 20 beam spills (5% of the planned dose - first column), 5 of 20 beam spills (25% of the planned dose - centre column) and 5 minutes post full-treatment (right column).

Figure 5 shows the 2D annihilation maps obtained during and after the simulated delivery of 5 Gy(RBE) for each beam type to the target volume within the skull phantom. Images in the first column correspond to data acquired during the first beam-off interval (i.e. after one spill), the centre column show images following 5 spills, and finally, the last column shows images acquired during the five minutes (300 seconds) immediately after the completion of the 20th (and final) spill.

The CNRs of the inside and outside of the proximal, distal and upper lateral boundaries of the SOBP images in Fig. 5 are listed in Table 5.

**Table 5:** Contrast-to-noise ratios (CNRs) corresponding to Fig. 5; the highest CNR value in each column is highlighted in bold.

	Proximal Edge			Lateral Edge			Distal Edge		
	1 spill	5 spills	5 min	1 spill	5 spills	5 min	1 spill	5 spills	5 min
^10^C	**156.47**	**332.99**	122.36	**120.26**	**286.72**	190.95	**125.38**	**338.18**	**335.90**
^11^C	9.7344	19.567	57.338	17.731	39.356	108.26	15.415	39.075	202.55
^12^C	4.5779	8.2736	12.979	11.034	19.265	21.380	2.9674	4.6759	6.8661
^15^O	28.398	79.051	**201.99**	72.384	133.94	**216.50**	53.606	85.169	233.24
^16^O	3.7561	8.7385	15.572	14.183	23.685	26.484	5.6323	9.5223	14.268

Positron annihilation maps acquired at different stages of the treatment process clearly demonstrate the potential improvements in range-verification QA that can be obtained with radioactive ion beams. Following a single spill, the boundaries of the SOBP are very clearly visible in the cases of ^10^C and ^15^O (Fig. 5(a,j)), due to their short half-lives (19.29 seconds and 122.24 seconds, respectively). The images from the ^10^C simulation also exhibit the the highest CNR values for all boundaries after both 1 and 5 spills (i.e. the delivery of 5% and 25% of the total planned dose) and 5 minutes after the delivery of the full treatment for the distal boundary. ^15^O also exhibits an excellent CNR following a 5 minute acquisition, demonstrating the best results for proximal and distal edge. The two stable beams produce images which are indistinct in comparison to any of the radioactive beam images. Due to its half life of 20.334 minutes, only a small number of positron annihilations resulting from decays of ^11^C are observed within the first beam-off period (Fig. 5(d)). The distal edge can be clearly seen, however the proximal edge is indistinct. Finally, in the long post-irradiation image acquisition (right column in Fig. 5), most primaries from the ^10^C and ^15^O beams have decayed, resulting in very similar high-contrast images. A substantial number of primaries have now decayed in the case of ^11^C, resulting in the emergence of a well-defined edges to the SOBP; it is expected that a ^11^C beam with a post-irradiation image acquisition of 20 minute or more will result in very high CNRs due to its longer half-life. By contrast, after a 5 minute acquisition, the distal and proximal edges of the SOBP remain indistinct in the case of ^12^C. ^16^O exhibits a more well-defined distal edge to its SOBP compared to ^12^C, however, the proximal edge is again poorly defined.

The images also demonstrate one of the key differences between the radioactive and stable beams. For radioactive beams, positron annihilations principally occur in the vicinity of the stopping point of the primary particle. The intensity of the decay radiation observed in a PET image is therefore proportional to the number of primary particles which have arrived at that particular depth. The energy weightings required to achieve a flat biological dose have a bias towards higher energies (since more deeply-penetrating high-energy particles also deposit an entrance dose which is added to the dose deposited by lower energy beams). Therefore, the distal edge of the SOBP can be expected to be much brighter than the proximal edge, as is clearly evident in the images from the radioactive beams. By contrast, the contribution of primary or target fragmentation, which is relatively minor for the radioactive beams, is the *only* source of positrons in the case of the stable beams, and positron-emitting fragmentation products are produced to a varying extent along the entire length of the beam path (see Supplementary Tables S3 and S4). Therefore, the stable beams exhibit a flatter (although not completely flat) activity distribution in the SOBP, and weaker contrast between the SOBP and the entrance region.

### Radiation dose to patients

Given the superiority of positron-emitting radioactive beams for intra- and post-treatment QA imaging, it is also important to consider whether or not the use of such beams would have any unintended side effects for the patient. From this perspective, the main difference for the patient is that an additional radiation dose will result from the use of a radioactive beam. The dose resulting from the decay of a positron-emitting radionuclide includes the kinetic energy of the positrons together with the 511 keV gamma photons resulting from their eventual annihilation; for a ^11^C beam, a 70 Gy(RBE) dose delivered to a 100 mm cubic treatment volume would require approximately 2.3 × 10^11^ particles, distributed throughout the treatment volume. This corresponds to an initial activity concentration of 1.3 MBq/cc, which is comparable to tissue concentrations of radiotracer which would be used in diagnostic ^11^C clinical PET imaging, and would deliver a biological dose‘ within the treatment volume of the order of 3–10 mSv. The additional dose rapidly falls off outside the treatment volume, and would be insignificant compared to the dose due to lateral scattering of particles.

## Conclusion

This work aimed to quantitatively evaluate the therapeutic potential of positron-emitting radioactive heavy ion beams; in particular, with regard to the relative biological effectiveness of the beams compared to their non-radioactive counterparts, the spatial distribution of the positron-emitting annihilations generated during and after irradiation of the target, and the incidental dose to the patient. Monte Carlo simulations of heavy ion therapy using a pseudo-clinical spread out Bragg peak constructed with positron-emitting radioactive beams of ^11^C, ^10^C and ^15^O as well as stable ^12^C and ^16^O were undertaken with the Geant4 toolkit.

The simulation physics model was validated through a comparison of depth-dose curves for monoenergetic ^11^C, ^12^C, ^15^O and ^16^O beams and relative yield estimations of the positron-emitting fragments produced within the build-up and the Bragg peak region with experimental data for ^12^C and ^16^O obtained from the HIMAC facility in Japan. The maximum difference between the location of maximum dose in the simulation and experimental data was 0.8 mm, while the maximum difference in mean relative yields of the secondary positron-emitting fragments was 2%.

The radiobiological effectiveness (RBE_10_) of each beam was calculated for an SOBP extending from depths of 78 to 138 mm in a skull phantom using the modified microdosimetric kinetic model (MKM). The RBE_10_ of the radioactive ion beams was found to be within one standard deviation of the corresponding non-radioactive ion beams for all energies, indicating that the therapeutic efficacy of such beams should be very similar to beams of the corresponding non-radioactive ion.

Finally, the additional dose to the patient resulting from the use of radioactive beams was estimated to determine whether it poses any unreasonable risk to the patient compared to the use of a stable ion beam. The additional dose was found to be comparable to that received during diagnostic clinical PET, and therefore negligible compared to the dose delivered to the target volume or surrounding tissues during the radiotherapy procedure.

In summary, positron-emitting radioactive heavy ions are approximately equivalent to the corresponding stable isotope with respect to expected therapeutic properties in heavy ion radiotherapy, while being greatly superior to non-radioactive beams in terms of the potential for accurately imaging the treatment volume during and after treatment. The substantial increase in positron yield offered by positron-emitting radioactive beams for the same biological effective dose will allow the boundaries of the spread out Bragg peak in a PET image to be unambiguously identified, making the use of positron-emitting radioactive ions a compelling choice for heavy ion therapy.

## Supplementary information

Dataset 1

## Data Availability

All data generated or analysed during this study are included in this published article (and its Supplementary Information Files) or are available from the corresponding author on reasonable request.
